# Temporal Dynamics and Evolution of SARS-CoV-2 Demonstrate the Necessity of Ongoing Viral Genome Sequencing in Ontario, Canada

**DOI:** 10.1128/mSphere.00011-21

**Published:** 2021-05-05

**Authors:** Calvin P. Sjaarda, Jennifer L. Guthrie, Samira Mubareka, Jared T. Simpson, Bettina Hamelin, Henry Wong, Leanne Mortimer, Robert Slinger, Andrew G. McArthur, Marc Desjardins, Allison McGeer, Tony Mazzulli, Katya Douchant, Danielle Brabant-Kirwan, Ramzi Fattouh, Aaron Campigotto, Samir N. Patel, Nahuel Fittipaldi, Robert I. Colautti, Prameet M. Sheth

**Affiliations:** a Department of Psychiatry, Queen’s University, Kingston, Ontario, Canada; b Queen’s Genomics Lab at Ongwanada (Q-GLO), Ongwanada Resource Centre, Kingston, Ontario, Canada; c Public Health Ontario, Toronto, Ontario, Canada; d Sunnybrook Health Science Centre, Division of Infectious Diseases, Toronto, Ontario, Canada; e Laboratory Medicine and Pathobiology, University of Toronto, Toronto, Ontario, Canada; f Ontario Institute for Cancer Research, Toronto, Ontario, Canada; g Department of Computer Science, University of Toronto, Toronto, Ontario, Canada; h Ontario Genomics Institute, Toronto, Ontario, Canada; i Division of Microbiology, Kingston Health Sciences Centre, Kingston, Ontario, Canada; j Division of Microbiology, Eastern Ontario Regional Laboratories, Ottawa, Ontario, Canada; k Department of Laboratory Medicine and Pathology, CHEO, Ottawa, Ontario, Canada; l Department of Pathology and Laboratory Medicine, University of Ottawa, Ottawa, Ontario, Canada; m Department of Pediatrics, University of Ottawa, Ottawa, Ontario, Canada; n Department of Biochemistry and Biomedical Sciences, McMaster University, Hamilton, Ontario, Canada; o M.G. DeGroote Institute for Infectious Disease Research, McMaster University, Hamilton, Ontario, Canada; p David Braley Centre for Antibiotic Discovery, McMaster University, Hamilton, Ontario, Canada; q Faculty of Medicine, University of Ottawa, Ottawa, Ontario, Canada; r Department of Microbiology, Mount Sinai Hospital, University Health Network, Toronto, Ontario, Canada; s Department of Cell and Systems Biology, University of Toronto, Toronto, Ontario, Canada; t Department of Biomedical & Molecular Sciences, Queen’s University, Kingston, Ontario, Canada; u Division of Microbiology, Health Sciences North, Sudbury, Ontario, Canada; v St. Michael’s Unity Health, Toronto, Ontario, Canada; w Hospital for Sick Children, Toronto, Ontario, Canada; x Biology Department, Queen’s University, Kingston, Ontario, Canada; y Department of Pathology and Molecular Medicine, Queen’s University, Kingston, Ontario, Canada; z Gastrointestinal Disease Research Unit, Kingston Health Sciences Center, Kingston, Ontario, Canada; University of Maryland School of Medicine

**Keywords:** COVID-19, epidemiology, G614D, genetics, infectious disease, PANGOLIN lineage, public health, SARS-CoV-2

## Abstract

Genome-wide variation in SARS-CoV-2 reveals evolution and transmission dynamics which are critical considerations for disease control and prevention decisions. Here, we review estimates of the genome-wide viral mutation rates, summarize current COVID-19 case load in the province of Ontario, Canada (5 January 2021), and analyze published SARS-CoV-2 genomes from Ontario (collected prior to 24 November 2020) to test for more infectious genetic variants or lineages. The reported mutation rate (∼10^−6^ nucleotide [nt]^−1^ cycle^−1^) for SARS-CoV-2 is typical for coronaviruses. Analysis of published SARS-CoV-2 genomes revealed that the G614 spike protein mutation has dominated infections in Ontario and that SARS-CoV-2 lineages present in Ontario have not differed significantly in their rate of spread. These results suggest that the SARS-CoV-2 population circulating in Ontario has not changed significantly to date. However, ongoing genome monitoring is essential for identification of new variants and lineages that may contribute to increased viral transmission.

## Abstract (precis)

Genome-wide variation in SARS-CoV-2 reveals evolution and transmission dynamics which are critical considerations for disease control and prevention decisions. Here, we review estimates of the genome-wide viral mutation rates, summarize current COVID-19 case load in the province of Ontario, Canada (5 January 2021), and analyze published SARS-CoV-2 genomes from Ontario (collected prior to 24 November 2020) to test for more infectious genetic variants or lineages.

## PERSPECTIVE

Severe acute respiratory syndrome coronavirus 2 (SARS-CoV-2), the causative agent of coronavirus disease 2019 (COVID-19), has had devasting consequences on human health and wellbeing (1, 2), health care systems (3), and the global economy (4). Numerous studies have demonstrated the immense value of time-resolved SARS-CoV-2 genome sequencing for tracing viral origin (5, 6), mutational dynamics (7, 8), and transmission properties (9, 10) to inform public health decision-making (11).

**FIG 1:**
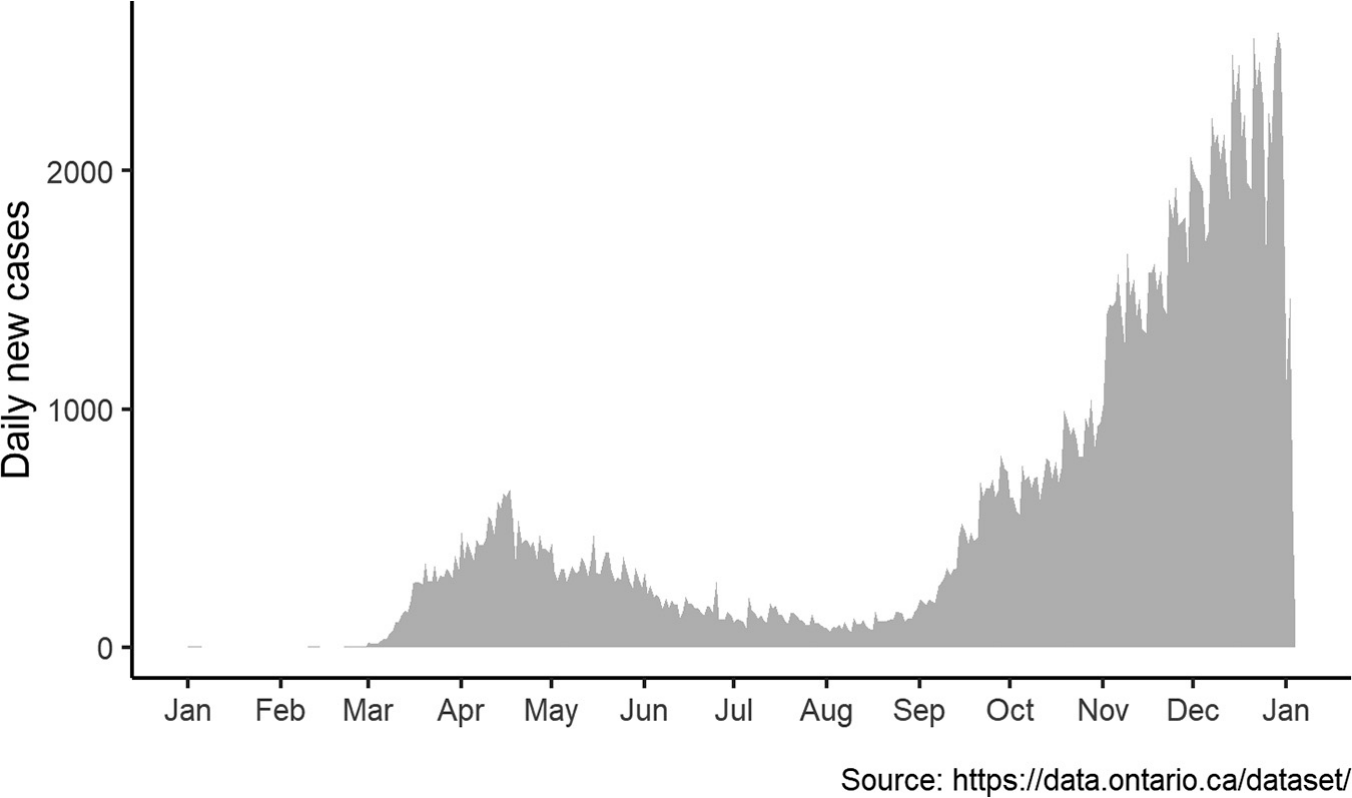
Daily COVID-19 case counts in Ontario, Canada, from 1 January 2020 until 5 January 2021 show surges of infections beginning in March and September 2020.

The first COVID-19 case in Ontario, Canada, was reported in January 2020, followed thereafter by a rapid increase in reported cases beginning in early March (Fig. 1). The number of new infections spiked in April followed by a significant decrease in new infections by mid-August, which coincided with heavy restrictions on everyday human activity, including physical distancing, limited social events, face mask requirements in public spaces, reduction of nonessential travel, and closing of schools and workplaces. However, only a month later the province saw a sharp rise in COVID-19 cases, signifying a second wave that has since surpassed the first wave in daily case counts (Fig. 1).

Potential contributors to the surge of infections in Ontario include changing behaviors of the host permitting the virus to be transmitted more easily or evolution of the pathogen enabling it to overcome barriers that had previously slowed its spread. To address concerns regarding the mutation of SARS-CoV-2 since its introduction into Ontario and the impact that these mutations may have on transmission efficiency of the virus, we have addressed the following questions. What is the published mutation rate of SARS-CoV-2 and how does it compare with other RNA viruses? Are there SARS-CoV-2 mutations (spike D614G) or lineages circulating in Ontario that may contribute to increased transmission of the virus which correlates with increases in infection rates?

## SARS-CoV-2 MUTATION RATE

**FIG 2:**
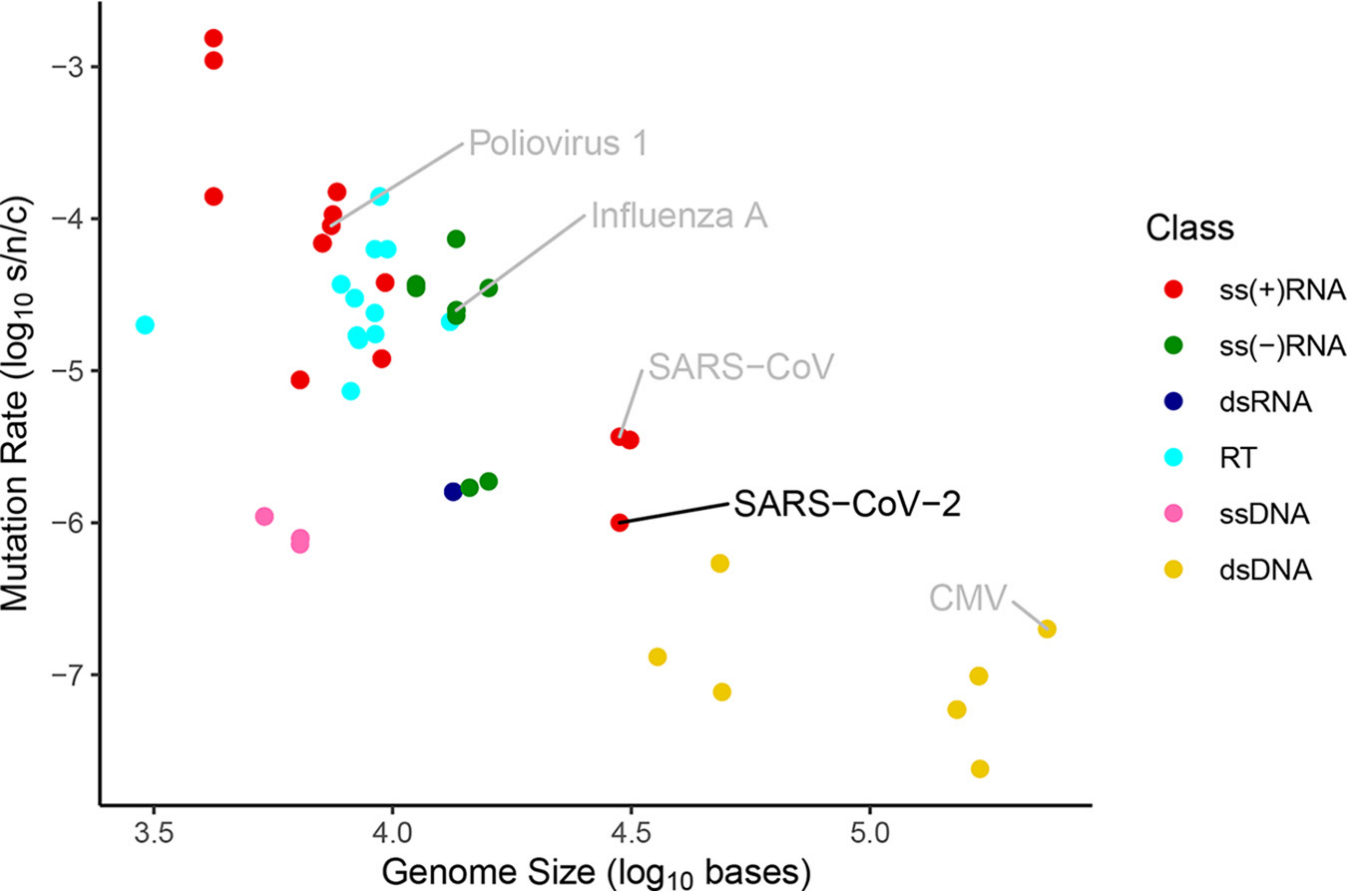
Viral mutation rates (substitutions per nucleotide site per cell infection [s/n/c]) depend on nucleotide type, number of strands, and genome size. Viral classes include positive single-stranded RNA [ss(+)RNA], negative single-stranded RNA [ss(−)RNA], double-stranded RNA (dsRNA), retrovirus (RT), single-stranded DNA (ssDNA), and double-stranded DNA (dsDNA), and comparison virus includes cytomegalovirus (CMV).

Viral evolution through genetic mutations enables viruses to increase virulence and transmissibility, escape host defenses, and infect new host species. There are several general trends that determine virus mutation rates (12–17) (Fig. 2). First, RNA viruses have mutation rates that are between 10 and 100 times higher than DNA viruses; a mutation rate of 10^−6^ to 10^−4^ substitutions per nucleotide (nt) per cell infection (s/n/c) is commonly observed in RNA viruses, compared to DNA viruses that mutate at a rate of ∼10^−8^ to 10^−6^ s/n/c (12). Second, single-stranded viruses (both DNA and RNA) have higher mutation rates than double-stranded viruses (13). Third, viral mutation rate inversely correlates with genome size (i.e., the larger the genome, the lower the overall mutation rate) (15, 18, 19). Single-stranded RNA viruses are typically characterized by the highest mutation rates, resulting in part from replication by a self-encoded RNA-dependent RNA polymerase that lacks proofreading activity. However, some single-stranded viruses, including coronaviruses, have exonuclease proofreading capability provided by the nonstructural protein (ExoN), which reduces mutations during replication (20) and may contribute to the maintenance of larger genomes observed in the group (21).

SARS-CoV-2 is a positive-sense, single-stranded RNA [ss(+)RNA] virus with a large genome that is typical of coronaviruses (∼29.9 kb) (22). It shares 80% nucleotide identity with SARS-CoV, the virus responsible for the SARS epidemic in 2003, and 55% nucleotide identity with Middle East respiratory syndrome coronavirus (MERS-CoV), described in 2012 (16). The mutation rate of SARS-CoV-2 (∼10^−6^ nt^−1^ cycle^−1^) (16) is low for an ss(+)RNA virus and has been reported to be similar to (7, 23, 24) or even lower than (25, 26) the mutation rate of other coronaviruses including SARS-CoV. The low mutation rate and high transmission rate of SARS-CoV-2 have resulted in distinct genetic lineages that are shared across large geographic regions. This global admixture of genomes that differ by <1% (27) has two important consequences for health care management. First, the low mutation rate reduces the probability of evolving resistance to therapeutic vaccines, such as those targeting the spike protein (7). Second, linking identical lineages in patient samples from around the world enables reconstruction of transmission pathways (5, 6, 11, 28).

## D614G MUTATION FREQUENCY

One of the most prevalent variants observed in SARS-CoV-2 sequenced genomes is the adenine-to-guanine nucleotide substitution at position 23403, a nonsynonymous mutation resulting in an amino acid substitution from aspartic acid to glycine at position 614 in the spike protein. The first D614G mutation was described in a viral genome sequence from China on 24 January 2020 followed by Germany on 28 January 2020 (29). This variant was found in 10% of globally published genomes by 1 March 2020, and became the most dominant form within a month (8). In the months that followed, hundreds of new lineages containing the D614G variant have been described. Countries that avoided a first wave of SARS-CoV-2 in January and February 2020, including most African and South American countries, report SARS-CoV-2 genotypes that are almost exclusively the G614 variant (29). The D614G amino acid change occurs in the spike protein but is outside the observed spike/ACE2 binding domain (amino acids 387 to 516) (30). The substitution from D to G reportedly enhances SARS-CoV-2 infectivity, competitive fitness, and transmission in primary human cells and animal models (31), which may contribute to the increasing variant frequency at multiple geographic locations, even those with an established D614 SARS-CoV-2 population (8, 32). In addition, viral loads are higher (demonstrated by lower reverse transcription-quantitative PCR (RT-qPCR) threshold cycle [*C_T_*] values) in COVID-19 patients infected with the G614 variant compared with the D614 variant (8, 32). Recently, at least one study has suggested that the second wave of COVID-19 cases may be a result of increased transmissibility of SARS-CoV-2 with the G614 variant (33). In contrast, another study found that D614G does not associate with significantly increased viral transmission and suggests that increases in D614 frequency are a demographic artifact due to a founder effect (34). To our knowledge, no study has shown that spike protein variants differ in virulence within human populations, and the potential impacts, if any, of D614G on the COVID-19 pandemic remain unclear (35).

**FIG 3:**
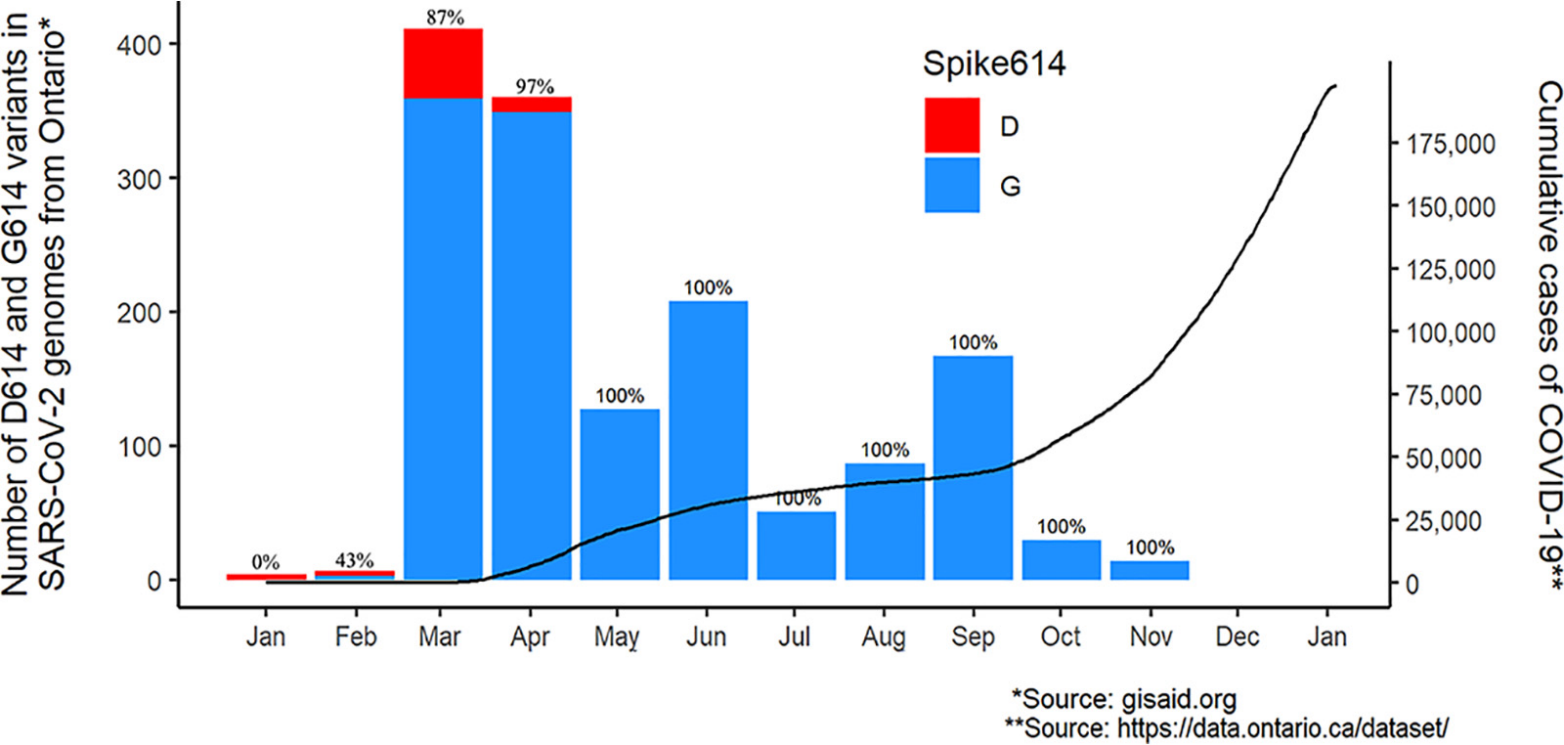
Total COVID-19 cases (line, right axis) and D614G variant distribution in published SARS-CoV-2 genomes (bars, left axis) from COVID-19 cases in Ontario from January 2020 to January 2021. Column labels represent the percentage of total sequences containing the G614 variant in each month.

Research, public health, and clinical laboratories in Ontario have been sequencing SARS-CoV-2 genomes since January 2020 and by 5 January 2021 shared a cumulative 1,743 genomes on the GISAID database (www.gisaid.org) (36). As this study investigated the temporal distribution of SARS-CoV-2 variants and lineages, the 78 samples in the GISAID database reporting a collection date with year only were removed from the analysis. In addition, samples collected on or after 24 November 2020, the first point prevalence analysis (to be published elsewhere), were also excluded, resulting in 1,466 viral genomes being included in this analysis. The SARS-CoV-2 genomes that were classified by GISAID as part of the G, GR, GH, or GV clade had the G614 variant, while all other clade classifications had the D614 variant (www.gisaid.org/references/statements-clarifications/clade-and-lineage-nomenclature-aids-in-genomic-epidemiology-of-active-hcov-19-viruses/). The earliest sequences (January and February) reported in Ontario have the wild-type sequence (D614), but most of the sequences reported during the first wave (March to June) are the mutant type (G614) (Fig. 3). In fact, the G614 variant accounts for 95.2% of all SARS-CoV-2 genomes reported in Ontario. Given that the G614 variant was already prevalent in Ontario at the beginning of the pandemic and that the D614 variant has not been detected since May, it is likely that the second wave has likewise been dominated by the G614 variant of SARS-CoV-2, suggesting that increased case numbers corresponding to the second wave of COVID-19 are likely unrelated to the spike protein variant. Our understanding of the circulating viral variants in Ontario, especially in rural areas, is limited by the available SARS-CoV-2 genome data on the GISAID server as the data demonstrate temporal bias (Fig. 3) and are geographically weighted to the greater Toronto area and eastern Ontario due to the laboratories sequencing SARS-CoV-2 being in these regions. As more viral genomes are sequenced and shared, this sampling bias may be reduced.

## FREQUENCY OF CIRCULATING LINEAGES

A dynamic nomenclature system based on a phylogenetic framework was proposed by Rambaut et al. (37) in July 2020 to help track the global spread of SARS-CoV-2 lineages. This nomenclature was quickly adopted by the international community, and a tool known as PANGOLIN (Phylogenetic Assignment of Named Global Outbreak LINeages) (https://github.com/hCoV-2019/pangolin) was developed to assign lineages of newly sequenced genomes. At the root of the SARS-CoV-2 pandemic is lineage A, which originated from China and disseminated globally. Arising from lineage A within China is lineage B, defined by two single nucleotide polymorphisms (SNPs), T8782C and C28144T (37). A number of other early lineages have been associated with specific geographies, including A.1 in the Washington State, USA, outbreak, B.1 in the Italian outbreak, and B.1.1, the major European lineage which was spread throughout the world (37). The variant causing the D614G mutation is characteristic of all SARS-CoV-2 genomes in B.1 and its descendant lineages (38). Many of these early lineages disseminated globally and are now present in most countries, with most local epidemics seeded by many independent introductions of the virus (39). Several lineages, including B.1.147 and B.1.114, have been characterized as Canadian SARS-CoV-2 lineages (https://cov-lineages.org/descriptions.html).

**FIG 4:**
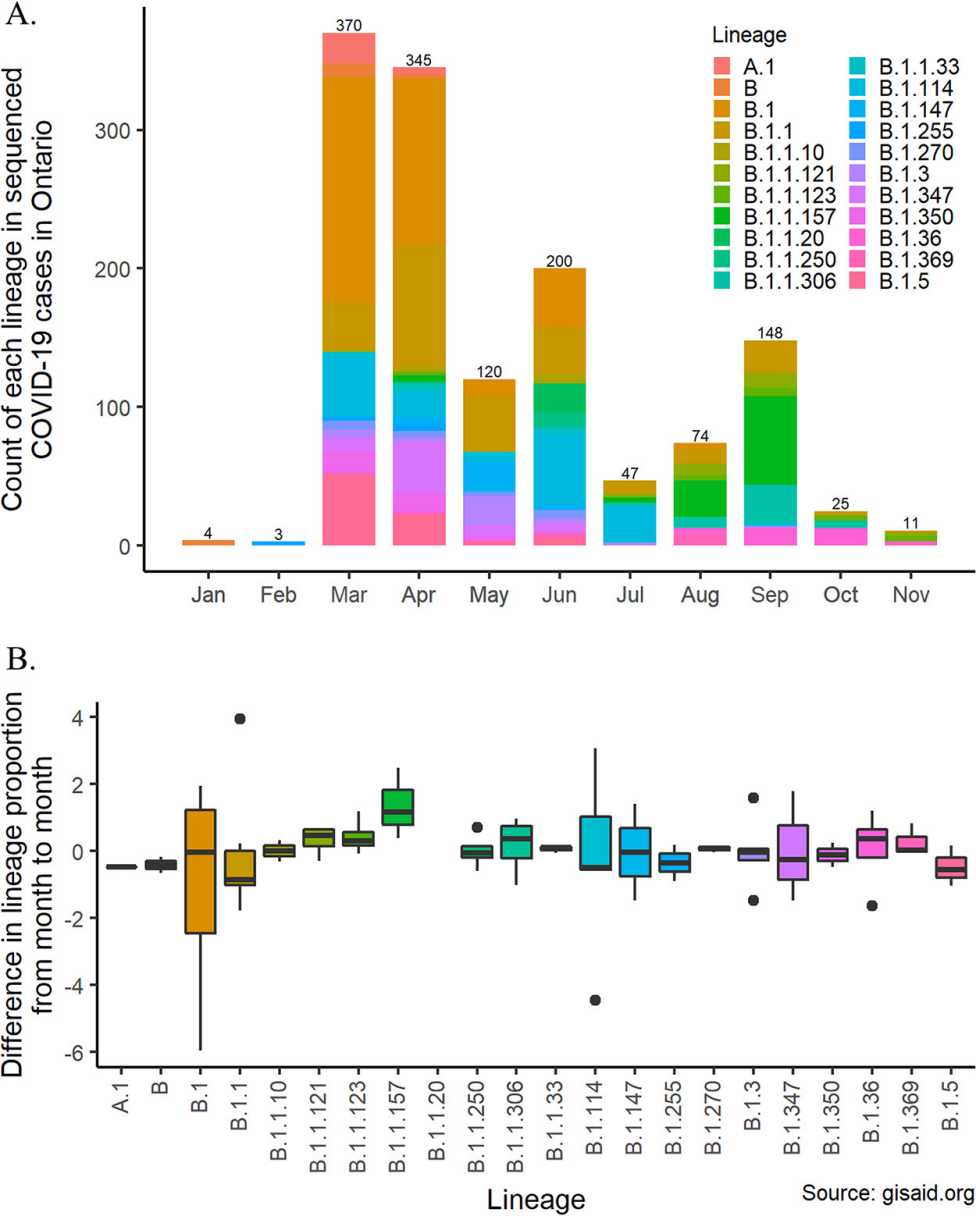
Distribution of major SARS-CoV-2 lineages sequenced from COVID-19 cases in Ontario. (A) Temporal distribution of major SARS-CoV-2 lineages of published genomes from January to December 2020. Column labels represent cumulative number of viral genomes for that month. (B) Box plot representing the monthly change in lineage prevalence (i.e., proportion of genomes) of each of 22 major SARS-CoV-2 lineages circulating in Ontario.

There are a total of 65 lineages identified in the 1,466 SARS-CoV-2 genomes published from COVID-19 cases in Ontario; however, only 22 lineages were observed ≥10 times and account for 91.9% of the published samples (Fig. 4A). No lineage dominates the more recent published genomes (Fig. 4A), and we detect no significant change in the monthly proportion of sequenced genomic lineages (Fig. 4B; F_20,52_ = 0.3098; *P* = >0.9; *R*^2^ = 0.106). Instead, lineage prevalence varies widely from month to month, consistent with stochastic effects of human activity such as a superspreader event that increases prevalence of a particular strain in 1 month only to be stamped out with contact tracing the next. However, this analysis is limited by the temporal and geographic biases discussed above. The novel lineage B.1.177 reported across Europe during the summer of 2020 was described in a single case in Ontario in September 2020, but no further cases with this lineage were found to date (40). Furthermore, lineage B.1.1.7 was recently described in the United Kingdom and preliminary characterization suggests that the new strain is significantly more transmissible (41). The presence of this lineage was confirmed in Ontario on 26 December 2020. Therefore, there is a clear need to sequence more SARS-CoV-2 genomes, more broadly sampled, especially from the second wave of infections, to increase statistical power for detecting more subtle variation among lineages in their contribution to the second and future waves of infections in Ontario.

## CONCLUSION

The COVID-19 pandemic has had a profound effect on the social and economic welfare of people and governments worldwide as they struggle to reduce the spread of the virus through implementation of comprehensive and aggressive public health measures. Our results contribute to our understanding of SARS-CoV-2, its reported mutation rate, and the variants and lineages that have been circulating in Ontario, Canada. SARS-CoV-2 has a genome size and mutation rate that are typical for coronaviruses and is reported to have a similar or lower mutation rate than SARS-CoV. Furthermore, Ontario’s circulating SARS-CoV-2 represents a relatively mixed genetic population resulting from multiple introductions and within-province spread. As discussed, preliminary evidence reportedly implicates the SARS-CoV-2 G614 variant as a more transmissible variant that may contribute to a second wave of the pandemic. However, this variant has been present in Ontario since the beginning of the pandemic, has been the dominant form of the virus in Ontario and in many other global regions, and is present in most sublineages that are currently circulating globally. We also detect no increased prevalence of a particular lineage that can account for the increase in cases in Ontario. It is possible that changes in human behavior are more likely explanations for the current wave of infections, perhaps due to relaxed control measures or declining compliance with existing measures. To slow transmission of COVID-19 and preserve health system capacity, the Province of Ontario implemented a second provincewide shutdown effective 26 December 2020. Sparse data limit our ability to detect differences in infection rate and highlight the need for Public Health Ontario Laboratory and academic research groups across Ontario to sequence and archive SARS-CoV-2 genomes from COVID-19 cases, particularly over the course of the second wave. Ongoing SARS-CoV-2 genomic surveillance, like the Canadian COVID Genomics Network (CanCOGeN) initiative, is essential to identify mutations that allow reconstruction of transmission pathways and detection of variants that affect transmissibility, virulence, or host mortality.
