# Supplementary material for: Temporal Dynamics and Evolution of SARS-CoV-2 Demonstrate the Necessity of Ongoing Viral Genome Sequencing in Ontario, Canada
Source: mSphere. 2021 May 5;6(3):e00011-21. doi: 10.1128/mSphere.00011-21 (PMC8103981; doi:10.1128/mSphere.00011-21)
Supplement: TABLE S1 [file mSphere.00011-21-st001.pdf]

We gratefully acknowledge the following Authors from the Originating laboratories responsible for obtaining the specimens, as well as the Submitting laboratories where the genome data were generated and shared via GISAID, on which this research is based.

All Submitters of data may be contacted directly via www.gisaid.org

Authors are sorted alphabetically.

Accession ID	Originating Laboratory	Submitting Laboratory	Authors
EPI_ISL_413014	Public Health Ontario Laboratory	Ontario Agency for Health Protection and Promotion (OAHPP)	Alireza Eshaghi, Samir N Patel, Jonathan B Gubbay, Vanessa G Allen, Christine Frantz, Aimin Li, Sandeep Nagra
EPI_ISL_413015	Public Health Ontario Laboratory	National Microbiology Laboratory	Shari Tyson, Anna Majer, Erika Landry, Morag Graham, Grace Seo, Philip Mabon, Natalie Knox, Adrian Zetner, Samira Mubareka, Rob Kozak, Jocelyne Lew, Darryl Falzarano, Gerdts Volker, Jonathan Gubbay, Stephanie Booth, Guillaume Poliquin, Tom Graefenhan, Matthew Gilmour, Nathalie Bastien, Yan Li, Timothy Booth
EPI_ISL_418322, EPI_ISL_418323, EPI_ISL_418324, EPI_ISL_418325, EPI_ISL_418326, EPI_ISL_418327, EPI_ISL_418328, EPI_ISL_418329, EPI_ISL_418330, EPI_ISL_418331, EPI_ISL_418332, EPI_ISL_418333, EPI_ISL_418334, EPI_ISL_418335, EPI_ISL_418336, EPI_ISL_418337, EPI_ISL_418338, EPI_ISL_418339, EPI_ISL_418340, EPI_ISL_418341, EPI_ISL_418342, EPI_ISL_418343, EPI_ISL_418344, EPI_ISL_418345, EPI_ISL_418346, EPI_ISL_418347, EPI_ISL_418348, EPI_ISL_418349, EPI_ISL_418350, EPI_ISL_418351, EPI_ISL_418352, EPI_ISL_418353, EPI_ISL_418354, EPI_ISL_418355, EPI_ISL_418356, EPI_ISL_418357, EPI_ISL_418358, EPI_ISL_418359, EPI_ISL_418360, EPI_ISL_418361, EPI_ISL_418362, EPI_ISL_418363, EPI_ISL_418364, EPI_ISL_418365, EPI_ISL_418366, EPI_ISL_418367, EPI_ISL_418368, EPI_ISL_418369, EPI_ISL_418370, EPI_ISL_418371, EPI_ISL_418372, EPI_ISL_418373, EPI_ISL_418374, EPI_ISL_418375, EPI_ISL_418376, EPI_ISL_418377, EPI_ISL_418378, EPI_ISL_418379, EPI_ISL_418380, EPI_ISL_418381, EPI_ISL_418382, EPI_ISL_418383, EPI_ISL_418384			
see above	Public Health Ontario Laboratories	Public Health Ontario Laboratories	Alireza Eshaghi, Samir N Patel, Jonathan B Gubbay, Vanessa G Allen, Christine Frantz, Aimin Li, Sandeep Nagra
EPI_ISL_425177	Public Health Ontario	Public Health Agency of Canada - National Microbiology Laboratory	Amrit S. Boese, Nikesh Tailor, Anders Leung, Joshua Quick, Shari Tyson, Morag Graham, Jonathan Audet, Natalie Knox, Darwyn Kobasa
EPI_ISL_450747	Sunnybrook Health Sciences Centre	Department of Laboratory Medicine and Molecular Diagnostics, Sunnybrook Health Sciences Centre	Jalees A. Nasir, Robert A. Kozak, Patryk Aftanas, Amogelang R. Raphenya, Kendrick M. Smith, Finlay Maguire, Hassaan Maan, Muhannad Alruwaili, Arinjay Banerjee, Hamza Mbareche, Brian P. Alcock, Natalie C. Knox, Karen Mossman, Bo Wang, Julian A. Hiscox, Andrew G. McArthur, Samira Mubareka
EPI_ISL_459866, EPI_ISL_459867, EPI_ISL_459868, EPI_ISL_459869, EPI_ISL_459871, EPI_ISL_459872, EPI_ISL_459873, EPI_ISL_459874, EPI_ISL_459875, EPI_ISL_459877, EPI_ISL_459878, EPI_ISL_459879, EPI_ISL_459880, EPI_ISL_459881, EPI_ISL_459882, EPI_ISL_459883, EPI_ISL_459884, EPI_ISL_459885, EPI_ISL_459886, EPI_ISL_459887, EPI_ISL_459888, EPI_ISL_459889, EPI_ISL_459890, EPI_ISL_459891, EPI_ISL_459892			
see above	Kingston Health Sciences Center	Queen's Genomics Lab at Ongwanada (Q-GLO)	Sjaarda CP, Rustom N, Huang D, Perez-Patrigeon S, Hudson ML, Wong H,Guan H, Ayub M, Soares CN, Colautti R, Evans GA, Sheth P
EPI_ISL_463970, EPI_ISL_463971, EPI_ISL_463972, EPI_ISL_463973, EPI_ISL_463974, EPI_ISL_463975, EPI_ISL_463976, EPI_ISL_463977, EPI_ISL_463978, EPI_ISL_463979, EPI_ISL_463980, EPI_ISL_463981, EPI_ISL_463982, EPI_ISL_463983, EPI_ISL_463984, EPI_ISL_463985, EPI_ISL_463986, EPI_ISL_463987, EPI_ISL_463988, EPI_ISL_463989, EPI_ISL_463990, EPI_ISL_463991, EPI_ISL_463992, EPI_ISL_463993, EPI_ISL_463994			
see above	Toronto Invasive Bacterial Diseases Network	McMaster University	Allison McGeer, Patryk Aftanas, Angel Li, Kuganya Nirmalarajah, Samira Mubareka, Andrew G. McArthur
EPI_ISL_463995, EPI_ISL_463996, EPI_ISL_463997, EPI_ISL_463998, EPI_ISL_463999, EPI_ISL_464000, EPI_ISL_464001, EPI_ISL_464002, EPI_ISL_464003, EPI_ISL_464004, EPI_ISL_464005, EPI_ISL_464006, EPI_ISL_464007, EPI_ISL_464008, EPI_ISL_464009, EPI_ISL_464010, EPI_ISL_464011, EPI_ISL_464012, EPI_ISL_464013, EPI_ISL_464014, EPI_ISL_464015, EPI_ISL_464016, EPI_ISL_464017, EPI_ISL_464018, EPI_ISL_464019, EPI_ISL_464020, EPI_ISL_464021, EPI_ISL_464022, EPI_ISL_464023, EPI_ISL_464024, EPI_ISL_464025, EPI_ISL_464026, EPI_ISL_464027, EPI_ISL_464028, EPI_ISL_464029, EPI_ISL_464030, EPI_ISL_464031, EPI_ISL_464032, EPI_ISL_464033, EPI_ISL_464034, EPI_ISL_464035, EPI_ISL_464036, EPI_ISL_464037, EPI_ISL_464038, EPI_ISL_464039, EPI_ISL_464040, EPI_ISL_464041, EPI_ISL_464042, EPI_ISL_464043, EPI_ISL_464044, EPI_ISL_464045, EPI_ISL_464046, EPI_ISL_464047, EPI_ISL_464048, EPI_ISL_464049, EPI_ISL_464050, EPI_ISL_464051, EPI_ISL_464052, EPI_ISL_464053, EPI_ISL_464054, EPI_ISL_464055, EPI_ISL_464056, EPI_ISL_464057, EPI_ISL_464058, EPI_ISL_464059, EPI_ISL_464060, EPI_ISL_464061, EPI_ISL_464062, EPI_ISL_464063, EPI_ISL_464064			
see above	Unity Health Toronto	Ontario Institute for Cancer Research	Ramzi Fattouh,Larissa M. Matukas,Mark Downing,Annette Gower,Karel Boissinot,Samira Mubareka,TIBDN,Ilinca Lungu,Bernard Lam,Jeremy Johns,Paul Krzyzanowski,Richard de Borja,Philip Zuzarte,Jared Simpson
EPI_ISL_494970	Dr. Tony Mazzulli Microbiologist-in-Chief	Dr. Jeff Wrana, Senior Investigator	Jeff Wrana, Jess Shen, Seda Barutcu, Kin Chan, Dan Trcka, Marie-Ming Aynaud, Javier Hernandez, Jessica Bourke, Christine Bruce, Bryn Hazlett, Laurence Pelletier, Sue Poutanen, Tony Mazzulli
EPI_ISL_495012	Dr. Tony Mazzulli Microbiologist-in-Chief	Dr. Jeff Wrana, Senior Investigator	Jeff Wrana, Jess Shen, Seda Barutcu, Kin Chan, Dan Trcka, Marie-Ming Aynaud, Javier Hernandez, Jessica Bourke, Christine Bruce, Bryn Hazlett, Laurence Pelletier, Sue Poutanen, Tony Mazzulli
EPI_ISL_495013	Dr. Tony Mazzulli Microbiologist-in-Chief	Dr. Jeff Wrana, Senior Investigator	Jeff Wrana, Jess Shen, Seda Barutcu, Kin Chan, Dan Trcka, Marie-Ming Aynaud, Javier Hernandez, Jessica Bourke, Christine Bruce, Bryn Hazlett, Laurence Pelletier, Sue Poutanen, Tony Mazzulli
EPI_ISL_526253, EPI_ISL_526254, EPI_ISL_526255, EPI_ISL_526256, EPI_ISL_526257, EPI_ISL_526258, EPI_ISL_526259, EPI_ISL_526260, EPI_ISL_526261, EPI_ISL_526262, EPI_ISL_526263, EPI_ISL_526264, EPI_ISL_526265, EPI_ISL_526266, EPI_ISL_526267, EPI_ISL_526268, EPI_ISL_526269, EPI_ISL_526270, EPI_ISL_526271, EPI_ISL_526272, EPI_ISL_526273, EPI_ISL_526274, EPI_ISL_526275, EPI_ISL_526276, EPI_ISL_526277, EPI_ISL_526278, EPI_ISL_526279, EPI_ISL_526280, EPI_ISL_526281, EPI_ISL_526282, EPI_ISL_526283, EPI_ISL_526284, EPI_ISL_526285, EPI_ISL_526286			
see above	Unity Health Toronto	Ontario Institute for Cancer Research	Ramzi Fattouh, Larissa M. Matukas, Mark Downing, Annette Gower, Karel Boissinot, Samira Mubareka, TIBDN, Ilinca Lungu, Bernard Lam, Jeremy Johns, Paul Krzyzanowski, Richard de Borja, Felicia Vincelli, Philip Zuzarte, Jared Simpson
EPI_ISL_529029, EPI_ISL_529030	Kingston Health Sciences Center	Queen's Genomics Lab at Ongwanada (Q-GLO)	Sjaarda CP, Rustom N, Huang D, Perez-Patrigeon S, Hudson ML, Wong H,Guan H, Ayub M, Soares CN, Colautti R, Evans GA, Sheth P
EPI_ISL_538321, EPI_ISL_538322, EPI_ISL_538323, EPI_ISL_538324, EPI_ISL_538325, EPI_ISL_538326, EPI_ISL_538327, EPI_ISL_538328, EPI_ISL_538329, EPI_ISL_538330, EPI_ISL_538331, EPI_ISL_538332, EPI_ISL_538333, EPI_ISL_538334, EPI_ISL_538335, EPI_ISL_538336, EPI_ISL_538337, EPI_ISL_538338, EPI_ISL_538339, EPI_ISL_538340, EPI_ISL_538341, EPI_ISL_538342, EPI_ISL_538343, EPI_ISL_538344, EPI_ISL_538345, EPI_ISL_538346, EPI_ISL_538347, EPI_ISL_538348, EPI_ISL_538349, EPI_ISL_538350, EPI_ISL_538351, EPI_ISL_538352, EPI_ISL_538353, EPI_ISL_538354, EPI_ISL_538355, EPI_ISL_538356, EPI_ISL_538357, EPI_ISL_538358, EPI_ISL_538359, EPI_ISL_538360, EPI_ISL_538361, EPI_ISL_538362, EPI_ISL_538363, EPI_ISL_538364, EPI_ISL_538365, EPI_ISL_538366, EPI_ISL_538367, EPI_ISL_538368, EPI_ISL_538369, EPI_ISL_538370, EPI_ISL_538371, EPI_ISL_538372, EPI_ISL_538373, EPI_ISL_538374, EPI_ISL_538375, EPI_ISL_538376, EPI_ISL_538377, EPI_ISL_538378, EPI_ISL_538379, EPI_ISL_538380, EPI_ISL_538381, EPI_ISL_538382, EPI_ISL_538383, EPI_ISL_538384, EPI_ISL_538385, EPI_ISL_538386			
see above	Kingston Health Sciences Centre / Queen's University	Ontario Institute for Cancer Research	Prameet M. Sheth, Calvin Sjaarda, Robert Colautti, Katya Douchant, Ilinca Lungu, Bernard Lam, Paul Krzyzanowski, Michael Laszloffy, Lawrence E Heisler, Richard de Borja, Jared T. Simpson
EPI_ISL_548684, EPI_ISL_548685, EPI_ISL_548686, EPI_ISL_548687, EPI_ISL_548688, EPI_ISL_548689, EPI_ISL_548690, EPI_ISL_548691, EPI_ISL_548692, EPI_ISL_548693, EPI_ISL_548694, EPI_ISL_548695, EPI_ISL_548696, EPI_ISL_548697, EPI_ISL_548698, EPI_ISL_548699, EPI_ISL_548700, EPI_ISL_548701, EPI_ISL_548702, EPI_ISL_548703, EPI_ISL_548704, EPI_ISL_548705, EPI_ISL_548706, EPI_ISL_548707, EPI_ISL_548708, EPI_ISL_548709, EPI_ISL_548710, EPI_ISL_548711, EPI_ISL_548712, EPI_ISL_548713, EPI_ISL_548714, EPI_ISL_548715, EPI_ISL_548716, EPI_ISL_548717, EPI_ISL_548718, EPI_ISL_548719, EPI_ISL_548720, EPI_ISL_548721, EPI_ISL_548722, EPI_ISL_548723, EPI_ISL_548724, EPI_ISL_548725, EPI_ISL_548726, EPI_ISL_548727, EPI_ISL_548728, EPI_ISL_548729, EPI_ISL_548730, EPI_ISL_548731, EPI_ISL_548732, EPI_ISL_548733, EPI_ISL_548734, EPI_ISL_548735, EPI_ISL_548736, EPI_ISL_548737, EPI_ISL_548738, EPI_ISL_548739, EPI_ISL_548740, EPI_ISL_548741, EPI_ISL_548742, EPI_ISL_548743, EPI_ISL_548744, EPI_ISL_548745, EPI_ISL_548746, EPI_ISL_548747, EPI_ISL_548748, EPI_ISL_548749, EPI_ISL_548750, EPI_ISL_548751, EPI_ISL_548752, EPI_ISL_548753, EPI_ISL_548754, EPI_ISL_548755, EPI_ISL_548756, EPI_ISL_548757, EPI_ISL_548758, EPI_ISL_548759, EPI_ISL_548760, EPI_ISL_548761, EPI_ISL_548762, EPI_ISL_548763, EPI_ISL_548764, EPI_ISL_548765, EPI_ISL_548766, EPI_ISL_548767, EPI_ISL_548768, EPI_ISL_548769, EPI_ISL_548770, EPI_ISL_548771, EPI_ISL_548772, EPI_ISL_548773, EPI_ISL_548774, EPI_ISL_548775, EPI_ISL_548776, EPI_ISL_548777, EPI_ISL_548778, EPI_ISL_548779, EPI_ISL_548780, EPI_ISL_548781, EPI_ISL_548782, EPI_ISL_548783, EPI_ISL_548784, EPI_ISL_548785, EPI_ISL_548786, EPI_ISL_548787, EPI_ISL_548788, EPI_ISL_548789, EPI_ISL_548790, EPI_ISL_548791, EPI_ISL_548792, EPI_ISL_548793, EPI_ISL_548794, EPI_ISL_548795, EPI_ISL_548796, EPI_ISL_548797, EPI_ISL_548798, EPI_ISL_548799, EPI_ISL_548800, EPI_ISL_548801, EPI_ISL_548802, EPI_ISL_548803, EPI_ISL_548804, EPI_ISL_548805, EPI_ISL_548806, EPI_ISL_548807, EPI_ISL_548808, EPI_ISL_548809, EPI_ISL_548810, EPI_ISL_548811, EPI_ISL_548812, EPI_ISL_548813, EPI_ISL_548814, EPI_ISL_548815, EPI_ISL_548816, EPI_ISL_548817, EPI_ISL_548818, EPI_ISL_548819, EPI_ISL_548820, EPI_ISL_548821, EPI_ISL_548822, EPI_ISL_548823, EPI_ISL_548824, EPI_ISL_548825, EPI_ISL_548826, EPI_ISL_548827, EPI_ISL_548828, EPI_ISL_548829, EPI_ISL_548830, EPI_ISL_548831, EPI_ISL_548832, EPI_ISL_548833, EPI_ISL_548834, EPI_ISL_548835, EPI_ISL_548836, EPI_ISL_548837, EPI_ISL_548838, EPI_ISL_548839, EPI_ISL_548840, EPI_ISL_548841, EPI_ISL_548842, EPI_ISL_548843, EPI_ISL_548844, EPI_ISL_548845, EPI_ISL_548846, EPI_ISL_548847, EPI_ISL_548848, EPI_ISL_548849, EPI_ISL_548850, EPI_ISL_548851, EPI_ISL_548852, EPI_ISL_548853, EPI_ISL_548854, EPI_ISL_548855, EPI_ISL_548856, EPI_ISL_548857, EPI_ISL_548858, EPI_ISL_548859, EPI_ISL_548860, EPI_ISL_548861, EPI_ISL_548862, EPI_ISL_548863, EPI_ISL_548864, EPI_ISL_548865, EPI_ISL_548866, EPI_ISL_548867, EPI_ISL_548868, EPI_ISL_548869, EPI_ISL_548870, EPI_ISL_548871, EPI_ISL_548872, EPI_ISL_548873, EPI_ISL_548874, EPI_ISL_548875, EPI_ISL_548876, EPI_ISL_548877, EPI_ISL_548878, EPI_ISL_548879, EPI_ISL_548880, EPI_ISL_548881, EPI_ISL_548882, EPI_ISL_548883, EPI_ISL_548884, EPI_ISL_548885, EPI_ISL_548886, EPI_ISL_548887, EPI_ISL_548888, EPI_ISL_548889, EPI_ISL_548890, EPI_ISL_548891, EPI_ISL_548892, EPI_ISL_548893, EPI_ISL_548894, EPI_ISL_548895, EPI_ISL_548896, EPI_ISL_548897, EPI_ISL_548898, EPI_ISL_548899, EPI_ISL_548900, EPI_ISL_548901, EPI_ISL_548902, EPI_ISL_548903, EPI_ISL_548904, EPI_ISL_548905, EPI_ISL_548906, EPI_ISL_548907, EPI_ISL_548908, EPI_ISL_548909, EPI_ISL_548910, EPI_ISL_548911, EPI_ISL_548912, EPI_ISL_548913, EPI_ISL_548914, EPI_ISL_548915, EPI_ISL_548916, EPI_ISL_548917, EPI_ISL_548918, EPI_ISL_548919, EPI_ISL_548920, EPI_ISL_548921, EPI_ISL_548922, EPI_ISL_548923, EPI_ISL_548924, EPI_ISL_548925, EPI_ISL_548926, EPI_ISL_548927, EPI_ISL_548928, EPI_ISL_548929, EPI_ISL_548930, EPI_ISL_548931, EPI_ISL_548932, EPI_ISL_548933, EPI_ISL_548934, EPI_ISL_548935, EPI_ISL_548936, EPI_ISL_548937, EPI_ISL_548938, EPI_ISL_548939, EPI_ISL_548940, EPI_ISL_548941			
see above	Public Health Ontario Laboratory	Public Health Ontario Laboratory	Vanessa G Allen, Philip Banh, Richard de Borja, Yao Chen, Alireza Eshaghi, Nahuel Fittipaldi, Christine Frantz, Jonathan B Gubbay, Jennifer L Guthrie, Lawrence Heisler, Esha Joshi, Michael Laszloffy, Aimin Li, Michael CY Li, Dean Maxwell, Sandeep Nagra, Samir N Patel, Heather Rilkoff, Jared Simpson, Karthikeyan Sivaraman, Yogi Sundaravadanam, Sarah Teatero, Andre Villegas, Sandra Zittermann
EPI_ISL_569949, EPI_ISL_569950, EPI_ISL_569951, EPI_ISL_569952, EPI_ISL_569953, EPI_ISL_569954, EPI_ISL_569955, EPI_ISL_569956, EPI_ISL_569957, EPI_ISL_569958, EPI_ISL_569959, EPI_ISL_569960, EPI_ISL_569961, EPI_ISL_569962, EPI_ISL_569963, EPI_ISL_569964, EPI_ISL_569965, EPI_ISL_569966, EPI_ISL_569967, EPI_ISL_569968, EPI_ISL_569969, EPI_ISL_569970, EPI_ISL_569971, EPI_ISL_569972, EPI_ISL_569973, EPI_ISL_569974, EPI_ISL_569975, EPI_ISL_569976, EPI_ISL_569977, EPI_ISL_569978, EPI_ISL_569979, EPI_ISL_569980, EPI_ISL_569981, EPI_ISL_569982, EPI_ISL_569983, EPI_ISL_569984, EPI_ISL_569985, EPI_ISL_569986, EPI_ISL_569987, EPI_ISL_569988, EPI_ISL_569989, EPI_ISL_569990, EPI_ISL_569991, EPI_ISL_569992, EPI_ISL_569993, EPI_ISL_569994, EPI_ISL_569995, EPI_ISL_569996, EPI_ISL_569997, EPI_ISL_569998, EPI_ISL_569999, EPI_ISL_570000, EPI_ISL_570001, EPI_ISL_570002,			

EPI_ISL_570003, EPI_ISL_570004, EPI_ISL_570005, EPI_ISL_570006, EPI_ISL_570007, EPI_ISL_570008, EPI_ISL_570009, EPI_ISL_570010, EPI_ISL_570011, EPI_ISL_570012, EPI_ISL_570013, EPI_ISL_570014, EPI_ISL_570015, EPI_ISL_570016, EPI_ISL_570017, EPI_ISL_570018, EPI_ISL_570019, EPI_ISL_570020, EPI_ISL_570021, EPI_ISL_570022, EPI_ISL_570023, EPI_ISL_570024, EPI_ISL_570025, EPI_ISL_570026

see above — Unity Health Toronto — Ontario Institute for Cancer Research — Ramzi Fattouh, Larissa M. Matukas, Yan Chen,Mark Downing, Trina Otterman, Karel Boissinot, Wai Sum Siu, Zhi Cui, Le Luu, Samira Mubareka, TIBDN, Ilinca Lungu, Bernard Lam, Jeremy Johns, Paul Krzyzanowski, Richard de Borja, Felicia Vincelli, Philip Zuzarte, Jared T. Simpson

EPI_ISL_586267, EPI_ISL_586268, EPI_ISL_586269, EPI_ISL_586270, EPI_ISL_586271, EPI_ISL_586272, EPI_ISL_586273, EPI_ISL_586274, EPI_ISL_586275, EPI_ISL_586276, EPI_ISL_586277, EPI_ISL_586278, EPI_ISL_586279, EPI_ISL_586280, EPI_ISL_586281, EPI_ISL_586282, EPI_ISL_586283, EPI_ISL_586284, EPI_ISL_586285, EPI_ISL_586286, EPI_ISL_586287, EPI_ISL_586288, EPI_ISL_586289, EPI_ISL_586290, EPI_ISL_586291, EPI_ISL_586292, EPI_ISL_586293, EPI_ISL_586294, EPI_ISL_586295, EPI_ISL_586296, EPI_ISL_586297, EPI_ISL_586298, EPI_ISL_586299, EPI_ISL_586300, EPI_ISL_586301, EPI_ISL_586302, EPI_ISL_586303, EPI_ISL_586304, EPI_ISL_586305, EPI_ISL_586306, EPI_ISL_586307, EPI_ISL_586308, EPI_ISL_586309, EPI_ISL_586310, EPI_ISL_586311, EPI_ISL_586312, EPI_ISL_586313, EPI_ISL_586314, EPI_ISL_586315, EPI_ISL_586316, EPI_ISL_586317, EPI_ISL_586318, EPI_ISL_586319, EPI_ISL_586320, EPI_ISL_586321, EPI_ISL_586322, EPI_ISL_586323, EPI_ISL_586324, EPI_ISL_586325, EPI_ISL_586326, EPI_ISL_586327, EPI_ISL_586328, EPI_ISL_586329, EPI_ISL_586330, EPI_ISL_586331, EPI_ISL_586332, EPI_ISL_586333, EPI_ISL_586334, EPI_ISL_586335, EPI_ISL_586336, EPI_ISL_586337, EPI_ISL_586338, EPI_ISL_586339, EPI_ISL_586340, EPI_ISL_586341, EPI_ISL_586342, EPI_ISL_586343, EPI_ISL_586344, EPI_ISL_586345, EPI_ISL_586346, EPI_ISL_586347, EPI_ISL_586348, EPI_ISL_586349, EPI_ISL_586350, EPI_ISL_586351, EPI_ISL_586352, EPI_ISL_586353, EPI_ISL_586354, EPI_ISL_586355, EPI_ISL_586356, EPI_ISL_586357, EPI_ISL_586358, EPI_ISL_586359, EPI_ISL_586360, EPI_ISL_586361, EPI_ISL_586362, EPI_ISL_586363, EPI_ISL_586364, EPI_ISL_586365, EPI_ISL_586366, EPI_ISL_586367, EPI_ISL_586368, EPI_ISL_586369, EPI_ISL_586370, EPI_ISL_586371, EPI_ISL_586372, EPI_ISL_586373, EPI_ISL_586374, EPI_ISL_586375, EPI_ISL_586376, EPI_ISL_586377, EPI_ISL_586378, EPI_ISL_586379, EPI_ISL_586380, EPI_ISL_586381, EPI_ISL_586382, EPI_ISL_586383, EPI_ISL_586384, EPI_ISL_586385, EPI_ISL_586386, EPI_ISL_586387, EPI_ISL_586388, EPI_ISL_586389, EPI_ISL_586390, EPI_ISL_586391, EPI_ISL_586392, EPI_ISL_586393, EPI_ISL_586394, EPI_ISL_586395, EPI_ISL_586396, EPI_ISL_586397, EPI_ISL_586398, EPI_ISL_586399, EPI_ISL_586400, EPI_ISL_586401, EPI_ISL_586402, EPI_ISL_586403, EPI_ISL_586404, EPI_ISL_586405, EPI_ISL_586406, EPI_ISL_586407, EPI_ISL_586408, EPI_ISL_586409, EPI_ISL_586410, EPI_ISL_586411, EPI_ISL_586412, EPI_ISL_586413, EPI_ISL_586414, EPI_ISL_586415, EPI_ISL_586416, EPI_ISL_586417, EPI_ISL_586418, EPI_ISL_586419, EPI_ISL_586420, EPI_ISL_586421, EPI_ISL_586422, EPI_ISL_586423, EPI_ISL_586424, EPI_ISL_586425, EPI_ISL_586426, EPI_ISL_586427, EPI_ISL_586428, EPI_ISL_586429, EPI_ISL_586430, EPI_ISL_586431, EPI_ISL_586432, EPI_ISL_586433, EPI_ISL_586434, EPI_ISL_586435, EPI_ISL_586436, EPI_ISL_586437, EPI_ISL_586438, EPI_ISL_586439, EPI_ISL_586440, EPI_ISL_586441, EPI_ISL_586442, EPI_ISL_586443, EPI_ISL_586444, EPI_ISL_586445, EPI_ISL_586446, EPI_ISL_586447, EPI_ISL_586448, EPI_ISL_586449, EPI_ISL_586450, EPI_ISL_586451, EPI_ISL_586452, EPI_ISL_586453, EPI_ISL_586454, EPI_ISL_586455, EPI_ISL_586456, EPI_ISL_586457, EPI_ISL_586458, EPI_ISL_586459, EPI_ISL_586460, EPI_ISL_586461, EPI_ISL_586462, EPI_ISL_586463, EPI_ISL_586464, EPI_ISL_586465, EPI_ISL_586466, EPI_ISL_586467, EPI_ISL_586468, EPI_ISL_586469, EPI_ISL_586470, EPI_ISL_586471, EPI_ISL_586472, EPI_ISL_586473, EPI_ISL_586474, EPI_ISL_586475, EPI_ISL_586476, EPI_ISL_586477, EPI_ISL_586478, EPI_ISL_586479, EPI_ISL_586480, EPI_ISL_586481, EPI_ISL_586482, EPI_ISL_586483, EPI_ISL_586484, EPI_ISL_586485, EPI_ISL_586486, EPI_ISL_586487, EPI_ISL_586488, EPI_ISL_586489, EPI_ISL_586490, EPI_ISL_586491, EPI_ISL_586492, EPI_ISL_586493, EPI_ISL_586494, EPI_ISL_586495, EPI_ISL_586496, EPI_ISL_586497, EPI_ISL_586498, EPI_ISL_586499, EPI_ISL_586500, EPI_ISL_586501, EPI_ISL_586502, EPI_ISL_586503, EPI_ISL_586504, EPI_ISL_586505, EPI_ISL_586506, EPI_ISL_586507, EPI_ISL_586508, EPI_ISL_591100, EPI_ISL_591101, EPI_ISL_591102, EPI_ISL_591103, EPI_ISL_591104, EPI_ISL_591105, EPI_ISL_591106, EPI_ISL_591107, EPI_ISL_591108, EPI_ISL_591109, EPI_ISL_591110, EPI_ISL_591111, EPI_ISL_591112, EPI_ISL_591113, EPI_ISL_591114, EPI_ISL_591115, EPI_ISL_591116, EPI_ISL_591117, EPI_ISL_591118, EPI_ISL_591119, EPI_ISL_591120, EPI_ISL_591121, EPI_ISL_591122, EPI_ISL_591123, EPI_ISL_591124, EPI_ISL_591125, EPI_ISL_591126, EPI_ISL_591127, EPI_ISL_591128, EPI_ISL_591129, EPI_ISL_591130, EPI_ISL_591131, EPI_ISL_591132, EPI_ISL_591133, EPI_ISL_591134, EPI_ISL_591135, EPI_ISL_591136, EPI_ISL_591137, EPI_ISL_591138, EPI_ISL_591139, EPI_ISL_591140, EPI_ISL_591141, EPI_ISL_591142, EPI_ISL_591143, EPI_ISL_591144, EPI_ISL_591145, EPI_ISL_591146, EPI_ISL_591147, EPI_ISL_591148, EPI_ISL_591149, EPI_ISL_591150, EPI_ISL_591151, EPI_ISL_591152, EPI_ISL_591153, EPI_ISL_591154, EPI_ISL_591155, EPI_ISL_591156, EPI_ISL_591157, EPI_ISL_591158, EPI_ISL_591159, EPI_ISL_591160, EPI_ISL_591161, EPI_ISL_591162, EPI_ISL_591163, EPI_ISL_591164, EPI_ISL_591165, EPI_ISL_591166, EPI_ISL_591167, EPI_ISL_591168, EPI_ISL_591169, EPI_ISL_591170, EPI_ISL_591171, EPI_ISL_591172, EPI_ISL_591173, EPI_ISL_591174, EPI_ISL_591175, EPI_ISL_591176, EPI_ISL_591177, EPI_ISL_591178, EPI_ISL_591179, EPI_ISL_591180, EPI_ISL_591181, EPI_ISL_591182, EPI_ISL_591183, EPI_ISL_591184, EPI_ISL_591185, EPI_ISL_591186, EPI_ISL_591187, EPI_ISL_591188, EPI_ISL_591189, EPI_ISL_591190, EPI_ISL_591191, EPI_ISL_591192, EPI_ISL_591193, EPI_ISL_591194, EPI_ISL_591195, EPI_ISL_591196, EPI_ISL_591197, EPI_ISL_591198, EPI_ISL_591199, EPI_ISL_591200, EPI_ISL_591201, EPI_ISL_591202, EPI_ISL_591203, EPI_ISL_591204, EPI_ISL_591205, EPI_ISL_591206, EPI_ISL_591207, EPI_ISL_591208, EPI_ISL_591209, EPI_ISL_591210, EPI_ISL_591211, EPI_ISL_591212, EPI_ISL_591213, EPI_ISL_591214, EPI_ISL_591215, EPI_ISL_591216, EPI_ISL_591217, EPI_ISL_591218, EPI_ISL_591219, EPI_ISL_591220, EPI_ISL_591221, EPI_ISL_591222, EPI_ISL_591223, EPI_ISL_591224, EPI_ISL_591225, EPI_ISL_591226, EPI_ISL_591227, EPI_ISL_591228, EPI_ISL_591229, EPI_ISL_591230, EPI_ISL_591231, EPI_ISL_591232, EPI_ISL_591233, EPI_ISL_591234, EPI_ISL_591235, EPI_ISL_591236, EPI_ISL_591237, EPI_ISL_591238, EPI_ISL_591239, EPI_ISL_591240, EPI_ISL_591241, EPI_ISL_591242, EPI_ISL_591243, EPI_ISL_591244, EPI_ISL_591245, EPI_ISL_591246, EPI_ISL_591247, EPI_ISL_591248, EPI_ISL_591249, EPI_ISL_591250, EPI_ISL_591251, EPI_ISL_591252, EPI_ISL_591253, EPI_ISL_591254, EPI_ISL_591255, EPI_ISL_591256, EPI_ISL_591257, EPI_ISL_591258, EPI_ISL_591259, EPI_ISL_591260, EPI_ISL_591261, EPI_ISL_591262, EPI_ISL_591263, EPI_ISL_591264, EPI_ISL_591265, EPI_ISL_591266, EPI_ISL_591267, EPI_ISL_591268, EPI_ISL_591269

see above — Toronto Invasive Bacterial Diseases Network — McMaster University — Allison McGeer, Patryk Aftanas, Hooman Derakhshani, Angel Li, Kuganya Nirmalarajah, Emily Panousis, Ahmed Draia, Jalees Nasir, Michael Surette, Samira Mubareka, Andrew G. McArthur

EPI_ISL_593481, EPI_ISL_593483, EPI_ISL_593486, EPI_ISL_593487, EPI_ISL_593489, EPI_ISL_593490, EPI_ISL_593491, EPI_ISL_593492, EPI_ISL_593493, EPI_ISL_593494, EPI_ISL_593495, EPI_ISL_593496, EPI_ISL_593497, EPI_ISL_593498, EPI_ISL_593499, EPI_ISL_593501, EPI_ISL_593502, EPI_ISL_593503, EPI_ISL_593504, EPI_ISL_593506, EPI_ISL_593507, EPI_ISL_593508, EPI_ISL_593509, EPI_ISL_593511, EPI_ISL_593512, EPI_ISL_593513, EPI_ISL_593514, EPI_ISL_593515, EPI_ISL_593517, EPI_ISL_593518, EPI_ISL_593519, EPI_ISL_593523, EPI_ISL_593524, EPI_ISL_593525, EPI_ISL_593526, EPI_ISL_593527, EPI_ISL_593528, EPI_ISL_593529, EPI_ISL_593531, EPI_ISL_593532, EPI_ISL_593533, EPI_ISL_593534, EPI_ISL_593536, EPI_ISL_593537, EPI_ISL_593538, EPI_ISL_593539, EPI_ISL_593540, EPI_ISL_593541, EPI_ISL_593543, EPI_ISL_593544, EPI_ISL_593545, EPI_ISL_593546, EPI_ISL_593547, EPI_ISL_593548, EPI_ISL_593549, EPI_ISL_593550, EPI_ISL_593551

see above — Eastern Ontario Regional Laboratory Association — McMaster University — Leanne Mortimer, Hooman Derakhshani, Emily Panousis, Ahmed Draia, Jalees Nasir, Robert Slinger, Andrew G. McArthur

EPI_ISL_609801, EPI_ISL_609802, EPI_ISL_609803, EPI_ISL_609804, EPI_ISL_609805, EPI_ISL_609806, EPI_ISL_609807, EPI_ISL_609808, EPI_ISL_609809, EPI_ISL_609810, EPI_ISL_609811, EPI_ISL_609812, EPI_ISL_609813, EPI_ISL_609814, EPI_ISL_609815, EPI_ISL_609816, EPI_ISL_609817, EPI_ISL_609818, EPI_ISL_609819, EPI_ISL_609820, EPI_ISL_609821, EPI_ISL_609822, EPI_ISL_609823, EPI_ISL_609824, EPI_ISL_609825, EPI_ISL_609826

see above — Unity Health Toronto — Ontario Institute for Cancer Research — Ramzi Fattouh, Larissa M. Matukas, Yan Chen,Mark Downing, Trina Otterman, Karel Boissinot, Wai Sum Siu, Zhi Cui, Le Luu, Samira Mubareka, TIBDN, Ilinca Lungu, Bernard Lam, Jeremy Johns, Paul Krzyzanowski, Richard de Borja, Felicia Vincelli, Philip Zuzarte, Jared T. Simpson

EPI_ISL_636907, EPI_ISL_636908, EPI_ISL_636909, EPI_ISL_636910, EPI_ISL_636911, EPI_ISL_636912, EPI_ISL_636913, EPI_ISL_636914, EPI_ISL_636915, EPI_ISL_636916, EPI_ISL_636917, EPI_ISL_636918, EPI_ISL_636919, EPI_ISL_636920, EPI_ISL_636921, EPI_ISL_636922, EPI_ISL_636923, EPI_ISL_636924, EPI_ISL_636925, EPI_ISL_636926, EPI_ISL_636927, EPI_ISL_636928, EPI_ISL_636929, EPI_ISL_636930, EPI_ISL_636931, EPI_ISL_636932, EPI_ISL_636933, EPI_ISL_636934, EPI_ISL_636935, EPI_ISL_636936, EPI_ISL_636937, EPI_ISL_636938, EPI_ISL_636939, EPI_ISL_636940, EPI_ISL_636941, EPI_ISL_636942, EPI_ISL_636943, EPI_ISL_636944, EPI_ISL_636945, EPI_ISL_636946, EPI_ISL_636947, EPI_ISL_636948, EPI_ISL_636949, EPI_ISL_636950, EPI_ISL_636951, EPI_ISL_636952, EPI_ISL_636953, EPI_ISL_636954, EPI_ISL_636955, EPI_ISL_636956, EPI_ISL_636957, EPI_ISL_636958, EPI_ISL_636959, EPI_ISL_639727, EPI_ISL_639728, EPI_ISL_671496, EPI_ISL_671497, EPI_ISL_671498, EPI_ISL_671499, EPI_ISL_671500, EPI_ISL_671501, EPI_ISL_671502, EPI_ISL_671503, EPI_ISL_671504, EPI_ISL_671505, EPI_ISL_671506, EPI_ISL_671507, EPI_ISL_671508, EPI_ISL_671509, EPI_ISL_671510, EPI_ISL_671511, EPI_ISL_671512, EPI_ISL_671513, EPI_ISL_671514, EPI_ISL_671515, EPI_ISL_671516, EPI_ISL_671517, EPI_ISL_671518, EPI_ISL_671519, EPI_ISL_671520, EPI_ISL_671521, EPI_ISL_671522, EPI_ISL_671523, EPI_ISL_671524, EPI_ISL_671525, EPI_ISL_671526, EPI_ISL_671527, EPI_ISL_671528, EPI_ISL_671529, EPI_ISL_671530, EPI_ISL_671531, EPI_ISL_671532, EPI_ISL_671533, EPI_ISL_671534, EPI_ISL_671535, EPI_ISL_671536, EPI_ISL_671537, EPI_ISL_671538, EPI_ISL_671539, EPI_ISL_671540, EPI_ISL_671541, EPI_ISL_671542, EPI_ISL_671543, EPI_ISL_671544, EPI_ISL_671545, EPI_ISL_671546, EPI_ISL_671547, EPI_ISL_671548, EPI_ISL_671549, EPI_ISL_671550, EPI_ISL_671551, EPI_ISL_671552, EPI_ISL_671553, EPI_ISL_671554, EPI_ISL_671555, EPI_ISL_671556, EPI_ISL_671557, EPI_ISL_671558, EPI_ISL_671559, EPI_ISL_671560, EPI_ISL_671561, EPI_ISL_671562, EPI_ISL_671563, EPI_ISL_671564, EPI_ISL_671565, EPI_ISL_671566, EPI_ISL_671567, EPI_ISL_671568, EPI_ISL_671569, EPI_ISL_671570, EPI_ISL_671571, EPI_ISL_671572, EPI_ISL_671573, EPI_ISL_671574, EPI_ISL_671575, EPI_ISL_671576, EPI_ISL_671577, EPI_ISL_671578, EPI_ISL_671579, EPI_ISL_671580, EPI_ISL_671581, EPI_ISL_671582, EPI_ISL_671583, EPI_ISL_671584, EPI_ISL_671585, EPI_ISL_671586, EPI_ISL_671587, EPI_ISL_671588, EPI_ISL_671589, EPI_ISL_671590, EPI_ISL_671591, EPI_ISL_671592, EPI_ISL_671593, EPI_ISL_671594, EPI_ISL_671595, EPI_ISL_671596, EPI_ISL_671597, EPI_ISL_671598, EPI_ISL_671599, EPI_ISL_671600, EPI_ISL_671601, EPI_ISL_671602, EPI_ISL_671603, EPI_ISL_671604, EPI_ISL_671605, EPI_ISL_671606

see above — Public Health Ontario Laboratory — Public Health Ontario Laboratory — Vanessa G Allen, Philip Banh, Richard de Borja, Yao Chen, Alireza Eshaghi, Nahuel Fittipaldi, Christine Frantz, Jonathan B Gubbay, Jennifer L Guthrie, Lawrence Heisler, Esha Joshi, Michael Laszloffy, Aimin Li, Michael CY Li, Dean Maxwell, Sandeep Nagra, Samir N Patel, Heather Rilkoff, Jared Simpson, Karthikeyan Sivaraman, Yogi Sundaravadanam, Sarah Teatero, Andre Villegas, Sandra Zittermann

EPI_ISL_671652, EPI_ISL_671653, EPI_ISL_671654, EPI_ISL_671655, EPI_ISL_671656, EPI_ISL_671657, EPI_ISL_671658, EPI_ISL_671659, EPI_ISL_671660, EPI_ISL_671661, EPI_ISL_671662, EPI_ISL_671663, EPI_ISL_671664, EPI_ISL_671665, EPI_ISL_671666, EPI_ISL_671667, EPI_ISL_671668, EPI_ISL_671669, EPI_ISL_671670, EPI_ISL_671671, EPI_ISL_671672

see above — Unity Health Toronto — Ontario Institute for Cancer Research — Ramzi Fattouh, Larissa M. Matukas, Yan Chen,Mark Downing, Trina Otterman, Karel Boissinot, Wai Sum Siu, Zhi Cui, Le Luu, Samira Mubareka, TIBDN, Ilinca Lungu, Bernard Lam, Jeremy Johns, Paul Krzyzanowski, Richard de Borja, Felicia Vincelli, Philip Zuzarte, Jared T. Simpson

EPI_ISL_698079, EPI_ISL_698080, EPI_ISL_698081, EPI_ISL_698082, EPI_ISL_698083, EPI_ISL_698084, EPI_ISL_698085, EPI_ISL_698086, EPI_ISL_698087, EPI_ISL_698088, EPI_ISL_698089, EPI_ISL_698090, EPI_ISL_698091, EPI_ISL_698092, EPI_ISL_698093, EPI_ISL_698094, EPI_ISL_698095, EPI_ISL_698096, EPI_ISL_698097, EPI_ISL_698098, EPI_ISL_698099, EPI_ISL_698100, EPI_ISL_698101, EPI_ISL_698102, EPI_ISL_698103, EPI_ISL_698104

see above — Unity Health Toronto — Ontario Institute for Cancer Research — Ramzi Fattouh, Larissa M. Matukas, Yan Chen,Mark Downing, Trina Otterman, Karel Boissinot, Le Luu, Samira Mubareka, TIBDN, Ilinca Lungu, Bernard Lam, Jeremy Johns, Paul Krzyzanowski, Richard de Borja, Felicia Vincelli, Philip Zuzarte, Jared T. Simpson

EPI_ISL_717723, EPI_ISL_717724, EPI_ISL_717725, EPI_ISL_717726, EPI_ISL_717727, EPI_ISL_717728, EPI_ISL_717729, EPI_ISL_717730, EPI_ISL_717731, EPI_ISL_717732, EPI_ISL_717733, EPI_ISL_717734, EPI_ISL_717735, EPI_ISL_717736, EPI_ISL_717737, EPI_ISL_717738, EPI_ISL_717739, EPI_ISL_717740, EPI_ISL_717741, EPI_ISL_717742, EPI_ISL_717743, EPI_ISL_717744, EPI_ISL_717745, EPI_ISL_717746, EPI_ISL_717747, EPI_ISL_717748, EPI_ISL_717749, EPI_ISL_717750, EPI_ISL_717751, EPI_ISL_717752, EPI_ISL_717753, EPI_ISL_717754, EPI_ISL_717755, EPI_ISL_717756, EPI_ISL_717757, EPI_ISL_717758, EPI_ISL_717759, EPI_ISL_717760, EPI_ISL_717761, EPI_ISL_717762, EPI_ISL_717763, EPI_ISL_717764, EPI_ISL_718105

see above — Kingston Health Sciences Centre and Queen's University — Ontario Institute for Cancer Research — Prameet M. Sheth,Calvin Sjaarda,Robert Colautti,Katya Douchant,Ilinca Lungu,Bernard Lam,Paul Krzyzanowski,Michael Laszloffy,Lawrence E. Heisler,Richard de Borja,Jared T. Simpson

EPI_ISL_729259, EPI_ISL_729260, EPI_ISL_729261, EPI_ISL_729262, EPI_ISL_729263, EPI_ISL_729264, EPI_ISL_729265, EPI_ISL_729266, EPI_ISL_729267, EPI_ISL_729268, EPI_ISL_729269, EPI_ISL_729270, EPI_ISL_729271, EPI_ISL_729272, EPI_ISL_729273, EPI_ISL_729274, EPI_ISL_729275, EPI_ISL_729276, EPI_ISL_729277, EPI_ISL_729278, EPI_ISL_729279, EPI_ISL_729280, EPI_ISL_729281, EPI_ISL_729282, EPI_ISL_729283, EPI_ISL_729284, EPI_ISL_729285, EPI_ISL_729286, EPI_ISL_729287, EPI_ISL_729288, EPI_ISL_729289, EPI_ISL_729290, EPI_ISL_729291, EPI_ISL_729292, EPI_ISL_729293, EPI_ISL_729294, EPI_ISL_729295, EPI_ISL_729296, EPI_ISL_729297, EPI_ISL_729298, EPI_ISL_729299, EPI_ISL_729300, EPI_ISL_729301, EPI_ISL_729302, EPI_ISL_729303, EPI_ISL_729304, EPI_ISL_729305, EPI_ISL_729306, EPI_ISL_729307, EPI_ISL_729308, EPI_ISL_729309, EPI_ISL_729310, EPI_ISL_729311, EPI_ISL_729312, EPI_ISL_729313, EPI_ISL_729314, EPI_ISL_729315, EPI_ISL_729316, EPI_ISL_729317, EPI_ISL_729318, EPI_ISL_729319, EPI_ISL_729320, EPI_ISL_729321, EPI_ISL_729322, EPI_ISL_729323, EPI_ISL_729324, EPI_ISL_729325, EPI_ISL_729326, EPI_ISL_729327, EPI_ISL_729328, EPI_ISL_729329, EPI_ISL_729330, EPI_ISL_729331, EPI_ISL_729332, EPI_ISL_729333, EPI_ISL_729334, EPI_ISL_729335

see above — Toronto Invasive Bacterial Diseases Network — McMaster University — Allison McGeer, Patryk Aftanas, Hooman Derakhshani, Angel Li, Kuganya Nirmalarajah, Emily Panousis, Ahmed Draia, Jalees Nasir, Michael Surette, Samira Mubareka, Andrew G. McArthur

EPI_ISL_751796, EPI_ISL_751797, EPI_ISL_751798 — Public Health Ontario Laboratory — Public Health Ontario Laboratory — Vanessa G Allen, Philip Banh, Richard de Borja, Yao Chen, Alireza Eshaghi, Nahuel Fittipaldi, Christine Frantz, Jonathan B Gubbay, Jennifer L Guthrie, Lawrence Heisler, Esha Joshi, Michael Laszloffy, Aimin Li, Michael CY Li, Dean Maxwell, Sandeep Nagra, Samir N Patel, Heather Rilkoff, Jared Simpson, Karthikeyan Sivaraman, Yogi Sundaravadanam, Sarah Teatero, Andre Villegas, Sandra Zittermann

EPI_ISL_755656, EPI_ISL_755657, EPI_ISL_755658, EPI_ISL_755659, EPI_ISL_755660, EPI_ISL_755661, EPI_ISL_755662, EPI_ISL_755663, EPI_ISL_755664, EPI_ISL_755665, EPI_ISL_755666, EPI_ISL_755667, EPI_ISL_755668, EPI_ISL_755669, EPI_ISL_755670, EPI_ISL_755671, EPI_ISL_755672, EPI_ISL_755673, EPI_ISL_755674, EPI_ISL_755675, EPI_ISL_755676, EPI_ISL_755677, EPI_ISL_755678, EPI_ISL_755679, EPI_ISL_755680, EPI_ISL_755681, EPI_ISL_755682, EPI_ISL_755683, EPI_ISL_755684, EPI_ISL_755685, EPI_ISL_755686, EPI_ISL_755687, EPI_ISL_755688, EPI_ISL_755689, EPI_ISL_755690, EPI_ISL_755691, EPI_ISL_755692, EPI_ISL_755693, EPI_ISL_755694, EPI_ISL_755695, EPI_ISL_755696, EPI_ISL_755697, EPI_ISL_755698, EPI_ISL_755699, EPI_ISL_755700, EPI_ISL_755701, EPI_ISL_755702, EPI_ISL_755703, EPI_ISL_755704, EPI_ISL_755705, EPI_ISL_755706, EPI_ISL_755707, EPI_ISL_755708, EPI_ISL_755709, EPI_ISL_755710, EPI_ISL_755711

see above	Toronto Invasive Bacterial Diseases Network	McMaster University	Allison McGeer, Patryk Aftanas, Hooman Derakhshani, Angel Li, Kuganya Nirmalarajah, Emily Panousis, Ahmed Draia, Jalees Nasir, Michael Surette, Samira Mubareka, Andrew G. McArthur
EPI_ISL_755712, EPI_ISL_755713, EPI_ISL_755714, EPI_ISL_755715, EPI_ISL_755716, EPI_ISL_755717, EPI_ISL_755718, EPI_ISL_755719, EPI_ISL_755720, EPI_ISL_755721, EPI_ISL_755722, EPI_ISL_755723, EPI_ISL_755724, EPI_ISL_755725, EPI_ISL_755726, EPI_ISL_755727, EPI_ISL_755728, EPI_ISL_755729, EPI_ISL_755730, EPI_ISL_755731, EPI_ISL_755732, EPI_ISL_755733, EPI_ISL_755734, EPI_ISL_755735, EPI_ISL_755736			
see above	Toronto Invasive Bacterial Diseases Network	McMaster University	David Richardson, Allison McGeer, Patryk Aftanas, Hooman Derakhshani, Angel Li, Kuganya Nirmalarajah, Emily Panousis, Ahmed Draia, Jalees Nasir, Michael Surette, Samira Mubareka, Andrew G. McArthur
EPI_ISL_755737, EPI_ISL_755738, EPI_ISL_755739, EPI_ISL_755740, EPI_ISL_755741, EPI_ISL_755742, EPI_ISL_755743, EPI_ISL_755744, EPI_ISL_755745, EPI_ISL_755746, EPI_ISL_755747, EPI_ISL_755748, EPI_ISL_755749, EPI_ISL_755750, EPI_ISL_755751, EPI_ISL_755752, EPI_ISL_755753, EPI_ISL_755754, EPI_ISL_755755, EPI_ISL_755756, EPI_ISL_755757, EPI_ISL_755758, EPI_ISL_755759, EPI_ISL_755760, EPI_ISL_755761, EPI_ISL_755762, EPI_ISL_755763, EPI_ISL_755764, EPI_ISL_755765, EPI_ISL_755766, EPI_ISL_755767, EPI_ISL_755768, EPI_ISL_755769, EPI_ISL_755770, EPI_ISL_755771, EPI_ISL_755772, EPI_ISL_755773, EPI_ISL_755774, EPI_ISL_755775, EPI_ISL_755776, EPI_ISL_755777, EPI_ISL_755778, EPI_ISL_755779, EPI_ISL_755780, EPI_ISL_755781, EPI_ISL_755782, EPI_ISL_755783, EPI_ISL_755784, EPI_ISL_755785, EPI_ISL_755786, EPI_ISL_755787, EPI_ISL_755788, EPI_ISL_755789, EPI_ISL_755790, EPI_ISL_755791, EPI_ISL_755792, EPI_ISL_755793, EPI_ISL_755794, EPI_ISL_755795, EPI_ISL_755796, EPI_ISL_755797, EPI_ISL_755798, EPI_ISL_755799, EPI_ISL_755800, EPI_ISL_755801, EPI_ISL_755802, EPI_ISL_755803, EPI_ISL_755804, EPI_ISL_755805, EPI_ISL_755806, EPI_ISL_755807, EPI_ISL_755808, EPI_ISL_755809, EPI_ISL_755810, EPI_ISL_755811, EPI_ISL_755812, EPI_ISL_755813, EPI_ISL_755814, EPI_ISL_755815, EPI_ISL_755816, EPI_ISL_755817, EPI_ISL_755818, EPI_ISL_755819, EPI_ISL_755820, EPI_ISL_755821, EPI_ISL_755822, EPI_ISL_755823, EPI_ISL_755824, EPI_ISL_755825, EPI_ISL_755826, EPI_ISL_755827, EPI_ISL_755828, EPI_ISL_755829, EPI_ISL_755830, EPI_ISL_755831, EPI_ISL_755832, EPI_ISL_755833, EPI_ISL_755834, EPI_ISL_755835, EPI_ISL_755836, EPI_ISL_755837, EPI_ISL_755838, EPI_ISL_755839, EPI_ISL_755840, EPI_ISL_755841, EPI_ISL_755842, EPI_ISL_755843, EPI_ISL_755844, EPI_ISL_755845, EPI_ISL_755846, EPI_ISL_755847, EPI_ISL_755848, EPI_ISL_755849, EPI_ISL_755850, EPI_ISL_755851, EPI_ISL_755852, EPI_ISL_755853, EPI_ISL_755854, EPI_ISL_755855, EPI_ISL_755856, EPI_ISL_755857, EPI_ISL_755858, EPI_ISL_755859, EPI_ISL_755860, EPI_ISL_755861, EPI_ISL_755862, EPI_ISL_755863, EPI_ISL_755864, EPI_ISL_755865, EPI_ISL_755866, EPI_ISL_755867, EPI_ISL_755868, EPI_ISL_755869, EPI_ISL_755870, EPI_ISL_755871, EPI_ISL_755872, EPI_ISL_755873, EPI_ISL_755874, EPI_ISL_755875, EPI_ISL_755876, EPI_ISL_755877, EPI_ISL_755878, EPI_ISL_755879, EPI_ISL_755880, EPI_ISL_755881, EPI_ISL_755882, EPI_ISL_755883, EPI_ISL_755884, EPI_ISL_755885, EPI_ISL_755886, EPI_ISL_755887, EPI_ISL_755888, EPI_ISL_755889, EPI_ISL_755890, EPI_ISL_755891, EPI_ISL_755892, EPI_ISL_755893, EPI_ISL_755894, EPI_ISL_755895, EPI_ISL_755896, EPI_ISL_755897, EPI_ISL_755898, EPI_ISL_755899, EPI_ISL_755900, EPI_ISL_755901, EPI_ISL_755902, EPI_ISL_755903, EPI_ISL_755904, EPI_ISL_755905, EPI_ISL_755906, EPI_ISL_755907, EPI_ISL_755908, EPI_ISL_755909, EPI_ISL_755910, EPI_ISL_755911, EPI_ISL_755912, EPI_ISL_755913, EPI_ISL_755914, EPI_ISL_755915, EPI_ISL_755916, EPI_ISL_755917, EPI_ISL_755918, EPI_ISL_755919, EPI_ISL_755920, EPI_ISL_755921, EPI_ISL_755922, EPI_ISL_755923, EPI_ISL_755924, EPI_ISL_755925, EPI_ISL_755926, EPI_ISL_755927, EPI_ISL_755928, EPI_ISL_755929, EPI_ISL_755930, EPI_ISL_755931, EPI_ISL_755932, EPI_ISL_755933, EPI_ISL_755934, EPI_ISL_755935, EPI_ISL_755936, EPI_ISL_755937, EPI_ISL_755938, EPI_ISL_755939			
see above	Toronto Invasive Bacterial Diseases Network	McMaster University	Allison McGeer, Patryk Aftanas, Hooman Derakhshani, Angel Li, Kuganya Nirmalarajah, Emily Panousis, Ahmed Draia, Jalees Nasir, Michael Surette, Samira Mubareka, Andrew G. McArthur
